# The Expression of Genes Related to Lipid Metabolism and Metabolic Disorders in Children before and after Hematopoietic Stem Cell Transplantation—A Prospective Observational Study

**DOI:** 10.3390/cancers13143614

**Published:** 2021-07-19

**Authors:** Wojciech Czogała, Małgorzata Czogała, Kinga Kwiecińska, Mirosław Bik-Multanowski, Przemysław Tomasik, Przemysław Hałubiec, Agnieszka Łazarczyk, Karol Miklusiak, Szymon Skoczeń

**Affiliations:** 1 Department of Pediatric Oncology and Hematology, University Children’s Hospital of Krakow, 30-663 Krakow, Poland; czogala@tlen.pl (W.C.); malgorzata.czogala@uj.edu.pl (M.C.); kinga.kwiecinska@uj.edu.pl (K.K.); 2 Department of Pediatric Oncology and Hematology, Faculty of Medicine, Jagiellonian University Medical College, 30-663 Krakow, Poland; 3 Department of Medical Genetics, Faculty of Medicine, Jagiellonian University Medical College, 30-663 Krakow, Poland; miroslaw.bik-multanowski@uj.edu.pl; 4 Department of Clinical Biochemistry, Faculty of Medicine, Jagiellonian University Medical College, 30-663 Krakow, Poland; p.tomasik@uj.edu.pl; 5 Student Scientific Group of Pediatric Oncology and Hematology, Jagiellonian University Medical College, 30-663 Krakow, Poland; przemyslawhalubiec@gmail.com (P.H.); agnieszka.lazarczyk@student.uj.edu.pl (A.Ł.); karolmiklusiak@gmail.com (K.M.)

**Keywords:** HSCT, lipids, dyslipidemia, insulin resistance, microarrays, expression, children

## Abstract

**Simple Summary:**

The increasing frequency of hematopoietic stem cell transplantation (HSCT) and average postprocedural survival time has improved the knowledge about long-term adverse effects of transplantation in adulthood; i.e., an increase in the incidence of systemic diseases, like cardiovascular diseases or metabolic syndromes, as well as various types of endocrine disorders. A screening test identifying children at high risk of metabolic complications of HSCT might be useful in this group of patients. The aim of our study was to investigate the microarray-determined expression of genes with known functions related to lipid metabolism and their correlation with laboratory and clinical parameters. The next phases of research should include preemptive management of lipid abnormalities based on the results of microarray analysis. This might be the basis of personalized therapy of lipid disorders in patients with dyslipidemia or abnormalities or key metabolic hormones, both before and after HSCT.

**Abstract:**

Metabolic disorders in children after hematopoietic stem cell transplantation (HSCT) are poorly characterized. However, it is known that dyslipidemia and insulin resistance are particularly common in these patients. We conducted a prospective study of 27 patients treated with HSCT to assess the possibility of predicting these abnormalities. We measured gene expressions using a microarray technique to identify differences in expression of genes associated with lipid metabolism before and after HSCT. In patients treated with HSCT, total cholesterol levels were significantly higher after the procedure compared with the values before HSCT. Microarray analysis revealed statistically significant differences in expressions of three genes, *DPP4*, *PLAG1*, and *SCD*, after applying the Benjamini–Hochberg procedure (p^BH^ < 0.05). In multiple logistic regression, the increase of *DPP4* gene expression before HCST (as well as its change between pre- and post-HSCT status) was associated with dyslipidemia. In children treated with HSCT, the burden of lipid disorders in short-term follow-up seems to be lower than before the procedure. The expression pattern of *DPP4* is linked with dyslipidemia after the transplantation.

## 1. Introduction

Hematopoietic stem cell transplantation (HSCT) is a procedure performed to treat various disorders, including hematopoietic malignancies, selected solid tumors, primary immunodeficiencies, and inborn errors of metabolism [1,2,3]. The range of its clinical applications continues to expand, particularly in children, leading to increased long-term survival associated with improved treatment of short-term complications [2,3,4,5]. Therefore, the next step is to improve the prediction and prevention of late morbidity and mortality of HSCT, preferably by early detection of patients at risk [6].

Therapeutic HSCT protocols used in individual indications are different, but the stem cell donor is indispensable for the transplantation procedure—it can be the patient (autologous transplantation) or an HLA-matched donor (allogeneic transplantation) [7]. Then the hematopoietic cells are administered to patient after prior conditioning. In most of cases, it involves high-dose chemotherapy or total-body irradiation with supralethal fractionated doses, causing bone marrow ablation [7]. Intensity of the treatment regimen, as well as toxicity of chemotherapeutic agents used during conditioning and immunosuppressive drugs (particularly cyclosporine (CsA) and glucocorticoids (GCs)), have significant impact on the general condition of patients and development of metabolic disorders, both by direct pharmacological effects and nutritional disturbances [1,8].

The increased use of HSCT and prolonged survival after the procedure improved our knowledge of its long-term adverse effects in adulthood [9,10]. An increase in the incidence of systemic conditions, such as cardiovascular diseases and metabolic syndromes, as well as various types of endocrine disorders, is seen in patients after HSCT compared with the general population [1,8,11,12]. Baker et al. showed that patients treated with HSCT had 3.65-fold higher risk of diabetes and approximately twofold higher risk of hypertension compared with controls [13]. Some of these disorders may develop several years after the procedure. Therefore, markers allowing for their prediction might lead to more effective prevention and improved quality of life of long-term HSCT survivors [1].

Metabolic disorders in children after HSCT are poorly characterized, and very limited data are available. Preliminary reports indicate that lipid abnormalities in patients after HSCT (occurring most often up to 6 months after transplantation) are key predictors of metabolic disorders in future [1,4,14,15]. Dyslipidemia and insulin resistance are particularly common in patients after HSCT, and more than 80% of them fulfill diagnostic criteria for dyslipidemia at least once within the first 100 days after transplantation [4,5,16], which, according to the established evidence, is associated with increased rates of late mortality [5,10,11,15,17]. To date, there are no high-quality guidelines on treatment of dyslipidemia in patients after HSCT. Current recommendations are based solely on expert opinions, and lipid-lowering treatments vary [4,5,16]. Although it is very likely that metabolic disorders may be associated with expressions of genes related to lipid metabolism, there is a gap in knowledge on the influence of transcriptome on metabolic parameters in children before and after HSCT.

Microarrays can obtain multiple data on various processes in an organism. The technique allows for simultaneous assessment of expressions of thousands of genes. The standard source of diagnostic material is peripheral blood, which is readily available. Mononuclears show high expression of genes involved in lipid homeostasis, and rapidly detect signals of its disturbance [18]. These genes’ expressions might be potential biomarkers of lipid-metabolism abnormalities.

Therefore, the aim of this work was to assess metabolic and clinical disorders related to lipid metabolism in children before HSCT and 6 months after the procedure. Another aim was to investigate expressions of genes with known functions related to lipid metabolism and their relationship with laboratory and clinical parameters. We also assessed whether lipid abnormalities or metabolic disorders could be predicted using baseline gene expressions in patients before HSCT treatment or their changes after the procedure.

## 2. Materials and Methods

### 2.1. Patients

**Figure 1:**
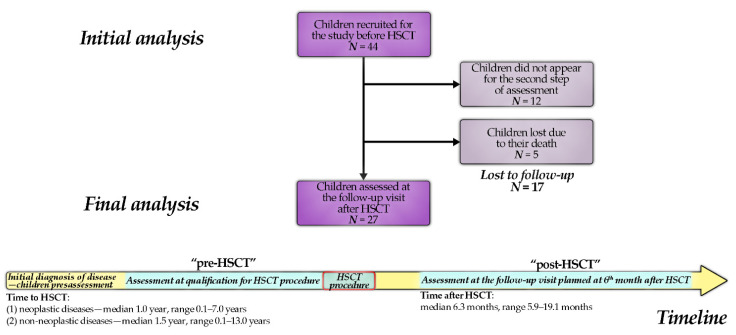
The study flowchart. The time range of each assessment step is shown.

We prospectively assessed 27 children (aged 1.5–18.0 years) in whom allogeneic HSCT was performed before the age of 18. The patients were referred to the Stem Cell Transplantation Center of the University Children’s Hospital in Krakow from June 2009 to October 2012 (indications for HSCT—see Table 1). Each patient was assessed twice: (1) before HSCT and (2) 6 months (median: 6.3 months) after the procedure (Figure 1). In all patients with malignancies (except for 1 patient), HSCT was performed in complete remission. On follow-up after 6 months, all children remained in remission with full donor chimerism. The conditioning regimens are summarized in Table 2, and the details of the HSCT procedure in Table 3.

The pre-HSCT and post-HSCT groups were estimated as 24 cases (providing large effect size of 0.80 and assuming α = 0.05 and test power 1−β = 0.95), therefore the recruitment was terminated at 110% of calculated value, in case some data were missing.

Exclusion criteria were: (1) lack of informed consent to participate in the study (provided by one of the parents and the patient if aged ≥ 16 years), (2) age > 18 years at the HSCT procedure, and (3) diagnosis of the disease significantly interfering with lipid metabolism. Only the children assessed both at the baseline and the follow-up visit were considered for final data analysis due to the paired character of the comparison.

The Permanent Ethical Committee for Clinical Studies of the Jagiellonian University Medical College approved the study design (KBET/96/B/2008 from 18 December 2008). Written informed consent to participate in study was obtained from the parents of all patients (and subjects aged ≥ 16 years). The study was conducted in accordance with the ethical principles set out in the Declaration of Helsinki [19].

### 2.2. Data Collection

Detailed demographical, clinical, and biochemical information was obtained at the time of enrollment and qualification of patients. Further data regarding transplantation: conditioning, complications, and their management were continually monitored and registered. The second step of data acquisition was planned at 6 months after HSCT (Figure 1).

#### 2.2.1. Laboratory Testing

Blood samples (1.5 mL) were collected in tubes containing EDTA, aprotinin (Bekcton-Dickinson; Franklin Lakes, UK) or in tubes without anticoagulant. The material was immediately delivered to the laboratory at +4 °C and centrifuged for 15 min at relative centrifugal force of 1590× *g*. Plasma and serum samples were stored at −80 °C until further analyses were performed.

Each participant underwent the standard oral glucose tolerance test (OGTT, 1.75 g of anhydrous glucose per every kilogram of body weight to a maximum of 75 g). Blood was drawn three times: on fasting (12 h, night-time), 60 min, and 120 min after glucose administration. The concentrations of triglycerides (TG), total cholesterol (TC), low-density lipoprotein cholesterol (LDL-C), and high-density lipoprotein cholesterol (HDL-C) in fasting blood samples were evaluated. Glucose, insulin, leptin, and soluble leptin receptor were measured for each OGTT time point (T_0_, T_60_, and T_120_). All area under the curve (AUC) values were calculated by application of the trapezoidal rule.

Glucose, TG, TC, LDL-C, and HDL-C levels were determined using Vitros 5.1 dry chemistry analyzer (Johnson & Johnson, United Kingdom; Department of Clinical Biochemistry, Polish-American Institute of Pediatrics). Insulin was measured by radioimmunometry (sensitivity: 1 µU/mL, inter-series precision: CV < 6.5%, intra-series precision: CV < 2.1%) (BioSource Company Europe S.A, Nivelles, Belgium). Leptin was measured using an enzyme-amplified sensitivity immunoassay technique (sensitivity: 0.1 ng/mL, inter-series precision: CV < 9.0%, intra-series precision: CV < 3.6%) (Biosource; Nivelles, Belgium), while soluble leptin receptor was measured using an enzyme immunoassay (EIA) (sensitivity: 0.04 ng/mL, inter-series precision: CV < 9.8%, intra-series precision: CV < 7.2%) (BioVendor Research and Diagnostic Products, Brno, Czech Republic).

Abnormal concentrations (i.e., indicative of dyslipidemia) were defined as: TG > 1.1 mmol/L (age 0–9 years) or >1.5 mmol/L (age 10–19 years), TC > 5 mmol/L, LDL-C > 3.2 mmol/L and HDL-C < 1 mmol/L [20]. Insulin resistance (IR) was determined using the HOMA-IR index (calculated as: glucose (mg/dL) × insulin (mIU/mL)/22.5), with a threshold value of > 2.5 as a criterion for IR [21,22,23].

#### 2.2.2. Anthropometric Measurements

All measurements were conducted by an anthropometrist. Body weight and height were measured with a stadiometer and a balanced scale, with precision levels of 0.1 kg and 0.1 cm, respectively. Waist circumference was assessed using standardized procedures according to the WHO guidelines (in the midpoint between lower costal margin and iliac crest in girls and at the level of umbilicus in boys). Body mass index (BMI), BMI (perc) and BMI (SDS) were calculated using a WHO calculator [24,25,26]. The results were compared to local and WHO-defined reference values. The parameters of body fat (total body water (TBW), extracellular water (ECW), lean body mass (LBM), body fat (kg) (BF (kg)), and BF (%)) were measured using bioimpedance and calculated according to the procedure proposed by Kushner and Scholler [27].

#### 2.2.3. Molecular Analysis (Microarrays)

Gene-expression assays were performed in the Department of Medical Genetics Chair of Pediatrics, Jagiellonian University Medical College, Krakow, Poland, laboratory with an international QC certificate (EMQN). Quality control was performed using principal component analysis (PCA), relative log expression (RLE), and normalized unscaled standard error (NUSE) plots.

Venous blood (0.3 mL) from each patient was used to determine gene expression. Leukocyte separation was performed using Ficoll density-gradient centrifugation. RNA was isolated using the RiboPure Blood Kit (Ambion, Life Technologies, Carlbad, CA, USA). RNA concentration was measured with a NanoDrop spectrophotometer (NanoDrop ND-1000; Thermoscientific, Carlsbad, CA, USA), and its quality was assessed with a 2100 Bioanalyzer (Agilent, Santa Clara, CA, USA). All procedures were performed according to the manufacturer’s protocol (GeneChip Whole Transcript sense Target Labeling Assay Manual, Version 4).

Microarray analysis was conducted using GeneChip Human Gene 1.0 ST Arrays (Affimetrix, Santa Clara, CA, USA) according to the manufacturer’s instructions. Gene expression was standardized in the RMA (robust multi-array average) procedure. Gene-expression data are presented as mean ± standard deviation (SD), representing the recorded signal intensity of the probes. It was assumed that the log_2_-transformed gene-expression levels were normally distributed, and the intergroup variance was of comparable magnitude. The difference in gene expression was calculated as follows: Δ = expression(post-HSCT) − expression(pre-HSCT), and Δ_rel_ = Δ/expression(pre-HSCT) × 100%, while Δ_mean_ (or Δ_mean(rel)_) was the arithmetical mean of calculated Δ (or Δ_rel_) values for a given gene.

Because the comparison showed differences in expressions of only 22 genes for the whole transcriptome (Table S1), the genes related to lipid disorders were manually selected based on literature data [28,29] and databases provided by GeneCards and KEGG [30]. Thus, a total of three genes associated with lipid metabolism was analyzed. A volcano plot was generated to show the changes in investigated gene expression compared to the overall shifts in expression pattern.

### 2.3. Statistical Analysis

The interval data are presented as mean ± SD, and categorical data as frequencies (*N*) and proportions (%). If any data were missing, the case was not included in the analysis for the given variable. Unpaired comparisons for categorical variables were conducted with the χ^2^ test (or two-tailed Fisher’s test if any expected number was <5 or group size *N* < 20). The McNemar’s test was used to compare categorical variables between pre-HSCT and post-HSCT. For the interval variables, the Student’s t-test (or Welch’s test in the case of variance non-homogeneity by Levene’s test) and the Student’s t-test for paired samples (in the pre-HSCT and post-HSCT comparisons) were run. If the Shapiro–Wilk’s test showed a non-normal distribution of the data, the non-parametric Mann–Whitney U test was conducted (or the Wilcoxon’s rank sum test in the case of the non-normal distribution of differences for paired data). Spearman’s correlation coefficient (r) was used to estimate the relationship between the interval variables.

Multivariate logistic regression and linear regression models incorporating the expression of individual genes were constructed in attempt to identify if genes were associated with dyslipidemia and insulin resistance and with abnormal parameters of lipid metabolism (respectively). A *p*-value below 0.05 was considered as significant. The Benjamini–Hochberg (BH) procedure was used to correct for multiple testing (assuming FDR = 0.05) and adjusted p^BH^ < 0.05 was considered as significant. All analyses were performed with Statistica 13.3 software (Statsoft Inc., Tulsa, OK, USA).

## 3. Results

### 3.1. Participants in HSCT and Control Groups

A total of 44 children underwent HSCT, and 27 of them (61%) were assessed at the follow-up visit. The remaining 17 children were unavailable due to death (five children) or failure to appear for reassessment after the planned period. Finally, a group of 27 children was considered in the analysis (Table 4). An average of 6.3 months elapsed between the pre-HSCT and post-HSCT visits (range: 5.9–19.1 months).

### 3.2. Metabolic and Anthropometric Characteristics

Before HSCT, insulin resistance was found in 9 (41%) children, and the features of dyslipidemia in 24 (86%) children. As assessed 6 months after transplantation, these proportions were slightly reduced for insulin resistance, which was still present in 6 patients (26%, including two new cases, *p* = 0.3), and remarkably lower for the features of dyslipidemia, which were present in 19 children (68%, including three new cases, *p*/p^BH^ = 0.001/0.006).

Significantly higher TC levels were found in the post-HSCT group compared to the pre-HSCT group (Table 5). In contrast, leptin levels at each measurement during the OGTT were higher in the pre-HSCT group (AUC for leptin: 30.1 ± 46.2 vs. 18.1 ± 35.5 ng/mL/h, p^BH^ = 0.02). Any features of dyslipidemia (defined as at least one of following: abnormal TC, HDL-C, LDL-C, or TG) were more frequent in the pre-HSCT than the post-HSCT group (86% vs. 68%, p^BH^ = 0.006). The additional comparison of metabolic parameters in the initial group of 44 children (pre-HSCT only) and between children that were treated with HSCT for neoplastic diseases versus non-neoplastic diseases is shown in Table S2 and Table S3, respectively.

Anthropometric parameters are presented in Tables S4 and S5.

### 3.3. Patterns of Gene Expression

In the whole transcriptome analysis, statistically significant differences of expression were found for 22 genes after the Benjamini–Hochberg procedure (p^BH^ < 0.05; 21 were downregulated and 1 was upregulated in the whole transcriptome). None of the cell-type specific genes had a different expression (i.e., relative frequencies of peripheral blood mononuclear cells were similar) (Table S1).

Taking FC into account, a remarkable expression change occurred for only one metabolism-associated gene; i.e., *DPP4* (Table 6). In a further search for the link between gene expression and metabolic parameters, we included all genes for which a significant difference between the groups (p^BH^ < 0.05) was demonstrated, to avoid missing a relevant relationship with gene expression, only due to adopting a rigid cut-off value for FC [31].

**Figure 2:**
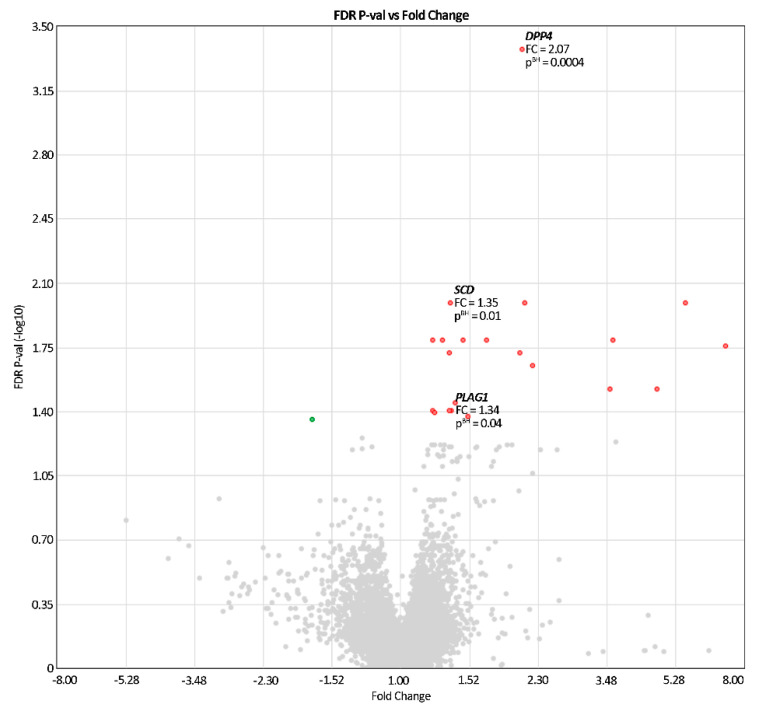
The volcano plot depicting differences in expression of 22 genes with significant expression differences between pre-HSCT and post-HSCT groups (colored dots) and in the whole transcriptome (gray dots).

Among the investigated genes related to lipid metabolism, significant shifts were observed in: *DPP4* (481.0 ± 1.5 vs. 230.7 ± 1.6, Δ_mean_ = 250.3 ± 233.0, p^BH^ = 0.0004), *PLAG1* (68.1 ± 1.4 vs. 50.6 ± 1.30, Δ_mean_ = 17.5 ± 28.4, p^BH^ = 0.016), and *SCD* (125.4 ± 1.4 vs. 93.1 ± 1.3, Δ_mean_ = 32.3 ± 54.0, p^BH^ = 0.010) (Table 6, Figure 2).

Comparison between children treated with HSCT for neoplastic diseases versus non-neoplastic diseases is shown in Table S6.

### 3.4. Changes in Gene Expression Related to Lipid-Metabolism Parameters

After corrections for multiple comparisons, a significant correlation was identified only for the relationship between *DPP4* expression and serum LDL-C when both were measured before HSCT (Spearman r = 0.51, p^BH^ = 0.03) (Table 7 and Table S7).

The expression levels of individual genes in children with metabolic abnormalities (insulin resistance and dyslipidemia) did not differ significantly from the patients without such abnormalities (corrected for multiple comparisons) (Table 8).

Significantly greater changes in *DPP4* expression were found in children who presented any laboratory features of dyslipidemia after HSCT compared to those who did not (Δ_mean_: 328.2 ± 204.0 vs. 46.0 ± 166.6, *p*/p^BH^ = 0.002/0.006). The same was true for relative changes in *DPP4* expression levels (Δ_mean(rel)_: 57.1 ± 36.0% vs. 3.8 ± 51.0%, *p*/p^BH^ = 0.002/0.006).

To assess the prospect of forecasting changes in the levels of lipid-metabolism parameters, as well as the presence of insulin resistance and dyslipidemia, multiple linear and logistic regression models were constructed. To limit the number of variables included for each model, only genes for which a significant correlation with the parameters of lipid metabolism was found (*p* < 0.05) were selected for the construction of linear regression models (additionally considering the age and sex of subjects, BMI WHO after the HSCT, and the type of an indication for HSCT). Following this approach, the model including sex, BMI WHO, and *DPP4* expression at pre-HSCT measurement explained 21% of variance in the LDL-C concentration after HSCT (R^2^_adj_ = 0.21, *p* = 0.04), although none of these variables predicted LDL-C independently (Table 9).

Multiple logistic regression for the comparison of gene expression or its change (Δ_mean_) with the occurrence of insulin resistance and dyslipidemia features was constructed using the expression of genes significantly associated with the latter in the t-test (*p* < 0.05), also incorporating the aforementioned covariables that were used in linear regression. *DPP4* expression (before HSCT or its change from pre- to post-HSCT, or its relative change) turned out to be an independent predictor of lipid-metabolism abnormalities (Table 10). Each model incorporating *DPP4* expression fitted data sufficiently (by the Hosmer–Lemeshow test; for each model, *p* = 0.1) (Table 10).

**Figure 3:**
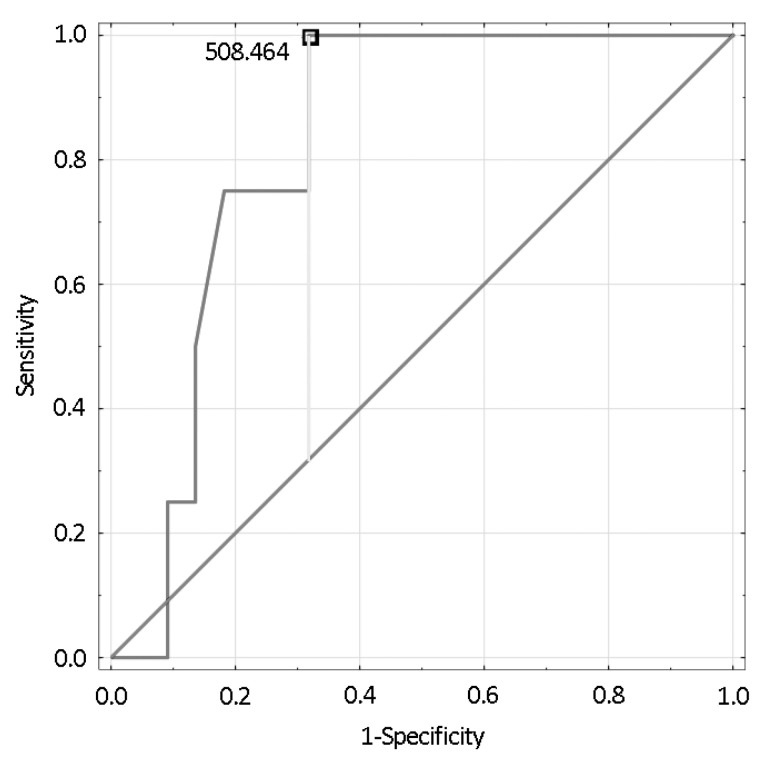
The ROC curve representing efficacy of *DDP4* expression before the HSCT in prediction of dyslipidemia after the HSCT.

The receiver operating characteristic (ROC) curve generated for *DPP4* expression before HSCT versus incidence of dyslipidemia after the transplantation identified, by the Youden index assessment, the expression of 508 units to be the cutoff value distinguishing subjects with (≥508) and without (<508) dyslipidemia after HSCT. The AUC for this model was 0.824, with *p* = 0.0001 (Figure 3).

## 4. Discussion

A remarkable finding of our study was the clear disparities in serum lipid profile between the pre-HSCT and post-HSCT subjects. Alterations in the expressions of genes related to lipid metabolism were found. Finally, we determined correlations of the selected gene expressions with lipid levels in children treated with HSCT, and settled the expression shift associated with features of dyslipidemia following HSCT.

### 4.1. Biochemical Parameters

Significant differences were found in serum lipid profiles before HSCT and 6 months after HSCT. A statistically significant increase in TC levels in patients after HSCT procedure, albeit without changes in levels of HDL-C, LDL-C, or HDL-C/TC ratio, shows the impact of HSCT on serum lipid profiles. Furthermore, any features of dyslipidemia were more frequent in the pre-HSCT group, and the reduction of lipid abnormalities in the post-HSCT group was statistically significant. These metabolic changes suggest a beneficial effect of HSCT in terms of lipid disturbances in children.

However, most of the HSCT recipients in our study had abnormal levels of at least one lipid parameter (regardless of the pre-HSCT/post-HSCT status), which is consistent with current literature data [14,16,17]. Similarly to our study, increases in individual lipid fractions were also seen in other papers [1,4,5,16]. In a recent study of similar design to our work and including a larger group of patients, Bis et al. reported increases in both TC and HDL-C after HSCT [1]. In another study, Premstaller et al. found that the median of baseline TC, LDL-C, and HDL-C levels before the first and subsequent transplantations were significantly higher in patients treated with autologous HSCT compared with those treated with allogeneic HSCT, while there was no significant difference in TG levels [4]. It is worth noting that GCs, often administered in high doses for the treatment of graft-versus-host disease, are well known to contribute to worsening of metabolic disturbances by promoting gluconeogenesis and lipogenesis, while increasing insulin resistance [14]. However, in our study, the median time from discontinuation of systemic GCs to the second assessment was 3.6 months. In contrast, Bis et al. did not observe multiple changes in the lipid profile during HSCT, similar to Cherian et al., who found that the prevalence of metabolic syndrome was not significantly different between patients treated with HSCT and controls [1,10]. On the other hand, Annaloro et al. showed that the prevalence of metabolic syndrome was twice higher than expected (compared with an age-adjusted general population cohort). However, it must be noted that this study only included adults [32].

Interestingly, the post-HSCT group showed significantly lower leptin levels than the pre-HSCT group, with higher (but non-significant) concentrations of leptin receptor levels in the post-HSCT group. The upregulation of leptin receptors seems to be triggered to maintain the lipid balance. Leptin, except for its metabolic impact, is well known for its immunomodulatory effects and plays a role in stimulation of hematopoiesis, so its low levels may be related to immunosuppression [33].

Insulin resistance is the core of the classic definition of metabolic syndrome, although further research is necessary to fully understand its pathophysiology and the gene–environment interactions that determine susceptibility [34]. Taskinen et al. reported that 52% of HSCT recipients had insulin resistance, including impaired glucose tolerance and type 2 diabetes [17], while Baker et al. calculated that patients after allogeneic HSCT were 3.65 times more likely to develop diabetes than healthy individuals [13]. In the study by Cherian et al., adults treated with allo-HSCT had increased insulin resistance compared with controls, and mean fasting and post-prandial glucose levels, HbA1c, and HOMA-IR were significantly higher in subjects older than 30 years of age than younger ones [10]. In our study, we found no differences in glucose or insulin levels between the pre-HSCT and post-HSCT groups, both fasting and after oral glucose administration. Moreover, a difference in insulin resistance rates between the pre-HSCT and post-HSCT groups was also non-significant.

### 4.2. Gene Expression

To the best of our knowledge, we were the first to analyze gene expression with the described association with lipid disorders in children treated with HSCT. The different levels of gene expression between pre-HSCT and post-HSCT groups could help to explain the changes in lipid metabolism in these patients.

Comparing the pre-HSCT and post-HSCT groups, after correction for multiple testing, we observed differences in expressions of the following genes: *DPP4*, *PLAG1* and *SCD*.

#### 4.2.1. *DPP4*

We found lower expression of *DPP4* in the post-HSCT group compared to the pre-HSCT group. However, among children with dyslipidemia features after HSCT, the reduction in *DPP4* expression was high, while in patients with no lipid metabolism abnormalities, the change was small. Importantly, the pre-HSCT expression itself was higher in patients with dyslipidemia compared with those without hyperlipidemia; however, the difference might have been an incidental result of multiple testing (*p* = 0.02, p^BH^ = 0.06).

The multiple linear regression analysis showed that the LDL-C levels after HSCT could be partially predicted by a simple model that incorporates sex, BMI, and *DPP4* expression. Unfortunately, the major part of heterogeneity was not explained by these factors, thus it could pose merely a supportive role in terms of predicting LDL-C abnormalities after transplantation.

Nonetheless, the most remarkable outcome of the analysis is that *DPP4* expression before HSCT (as well as its Δ_mean_ and Δ_mean(rel)_) could be used to anticipate the presence of dyslipidemia in children after transplantation. This feature was independent of main variables that could potentially interfere with lipid metabolism (i.e., sex, age, BMI, or underlying disease that was the indication for HSCT). Therefore, assessment of *DPP4* expression seems to be to a promising tool to drive clinical decisions regarding the proactive lipid-lowering treatment, or even treatment with *DPP4* inhibitors, which would obviously require careful, targeted research.

According to the GeneCard database, the dipeptidyl peptidase 4 (DPP4, named also CD26) is a serine exopeptidase with a dipeptidyl peptidase activity (cleaving peptides in the circulation, including chemokines, mitogenic growth factors, neuropeptides, and peptide hormones), additionally acting as a positive regulator of T-cell coactivation, enhancing cell proliferation when overexpressed [30]. Elevated liver expression of *DPP4* may promote non-alcoholic fatty liver disease and insulin resistance. The mechanism incorporates a decrease in active glucagon-like peptide 1 level, but also a direct auto- and paracrine effect of DPP4 on hepatic insulin signaling [35]. Unequivocal evidence for the clinical importance of DPP4 stems from the fact that its inhibitors are well acknowledged in pharmacotherapy of diabetes, helping to control the risk of atherosclerosis by reducing LDL-C levels, increasing HDL-C levels, and lowering blood pressure [36,37]. Moreover, they drive polarization of liver macrophages toward the M2 type, alleviating inflammatory processes and reducing insulin resistance [38].

Most of the currently available data regarding *DPP4* expression was derived from adult studies. Turcot et al. investigated the influence of methylation and expression of the *DPP4* gene in omental cells on the lipid metabolism. According to their observations in a group of 92 obese premenopausal women, *DPP4* expression correlated (r = 0.25) with plasma HDL-C/TC ratio [39]. In a cohort of 451 patients (median age 56 years), correlations between *DPP4* and fasting glucose (r = 0.218), insulin (r = 0.196), HOMA-IR (r = 0.210), and TG (r = 0.201) levels were shown [40]. A similar study was conducted in a group of 93 non-obese patients with type 2 diabetes. Plasma DPP4 levels were associated with LDL-C, fasting glucose, intra-abdominal adiposity, and upper-limb subcutaneous adipose tissue [41].

Association of *DPP4* expression with multiple metabolism-related parameters is well known. On the other hand, its influence depends on the population of concern. Our important finding is that *DPP4* expression could forecast dyslipidemia after HSCT, though it was not associated with insulin resistance.

#### 4.2.2. *PLAG1*

The expression of *PLAG1* was lower in the post-HSCT group. However, despite this difference, it was not associated with any metabolic parameter before or after HSCT.

The *PLAG1* zinc finger (*PLAG1*) gene encodes the protein functioning as a transcription factor responsible for upregulation and activation of target genes, such as *IGF2* or *IGFR1*, leading to uncontrolled cell proliferation. Recent reports indicated that it plays an important role in the development of obesity [30]. Certain target genes upregulated by PLAG1 influence glucose and lipid homeostasis; e.g., insulin-like growth factor 2 (IGF2), and are capable of reducing blood glucose levels, increasing the number of lipid droplets and free cholesterol content in murine liver cells, and upregulating 3-hydroxy-3-methylglutaryl-CoA reductase—the key enzyme of the cholesterol biosynthesis pathway [42,43]. Indirectly, the elevated expression of *PLAG1* may increase the concentration of lipid fractions in the serum.

Kadakia et al. studied cord blood of healthy infants, comparing genome methylation with concentrations of leptin. According to their observations, an increase in *PLAG1* methylation by 0.01 β value causes a decrease in leptin concentration by 9.4% [44]. This suggests that *PLAG1* influences the metabolic profile from the early stages of life, when regulation through IGF2 pathways plays an important role.

Hypothetically, we might have not found any association of *PLAG1* expression with laboratory and clinical variables in our subjects because the influence of this transcription factor was tampered. For example, its main molecular targets were potentially blocked through other mechanisms (i.e., *IGFR1* expression was reduced after HSCT, *p* < 0.05, though p^BH^ > 0.05).

#### 4.2.3. *SCD*

Pre-HSCT children had much higher *SCD* expression than after the transplantation, although like for *PLAG1*, the difference seemed not to influence the metabolic profile.

The stearoyl-CoA desaturase (*SCD*) gene encodes an enzyme involved in fatty acid biosynthesis, primarily the synthesis of oleic acid. The protein belongs to the fatty acid desaturase family and is an integral membrane protein located in the endoplasmic reticulum [30]. It regulates the expression of enzymes involved in lipogenesis (membrane phospholipids, cholesterol esters, and triglycerides) and mitochondrial fatty acid oxidation [30].

According to established research data, SCD is required for effective synthesis of TG and formation of adiposity [45]. Morcillo et al. suggested, that *SCD* methylation levels correlate negatively with free fatty acids and HOMA-IR [46]. Recently, reports of its beneficial effect were published. Oshima et al. showed that the *SCD* gene helps to maintain human β-cell function and protects them from lipotoxicity. *SCD* silencing induced markers of inflammation and endoplasmic reticulum stress, and the treatment with oleate or palmitoleate (both SCD products) reversed these abnormalities [47].

However, it should be stipulated that reduced *SCD* expression in patients before HSCT may not contribute to metabolic disorders after HSCT—at least we did not find any such relationship. Possibly, the observed high expression of *SCD* resulted from stress induced by HSCT itself, as the SCD counteracts cellular damage [48].

### 4.3. Study Limitations

The main limitation of our work was the small group of children included in the analysis. However, we obtained prospective outcomes in concordance with the results from other studies. The other concern was raised about the heterogeneity of the studied group; i.e., children with HSCT due to both the malignant and non-malignant disease. In terms of the main finding of our study (the predictive value of *DPP4* expression), we showed that this factor is not the determinant of the outcome, thus it does not reduce credibility of the results. We evaluated the changes in the expression profile of genes involved in lipid metabolism and proposed how they influence it. We have introduced a simple and patient-oriented method to create a prognostic model for metabolic abnormalities that may occur after HSCT. The results might require further confirmation on larger groups of subjects.

## 5. Conclusions

This prospective work demonstrated that in children after HSCT, in a relatively short-term follow-up of 6 months, the burden of lipid disorders tends to decrease. This remains in agreement with the limited data available from earlier research in the field. Still, among those patients are subjects particularly susceptible to sustain, or develop, dyslipidemia.

Analysis of transcriptome revealed that the expression patterns of some genes were strictly interconnected with abnormal lipid levels both before and after the transplantation. Knowledge about such relationships may become a cornerstone for future personalized therapy of lipid disorders in this unique group of patients; e.g., by means of designing drugs targeting products of genes with harmful effects (or enhancing those with a beneficial impact).

Finally, we proposed a feasible, microarray-based method to screen children planned for HSCT to assess their risk of metabolic abnormalities after the procedure. Assessment of gene expressions before HSCT or its change from pre-HSCT to post-HSCT would allow us to anticipate the incidence of dyslipidemia. Therefore, the next step of research should cover the idea of preemptive management directed by the results of microarray analysis, to avoid the persistence of lipid-metabolism disturbances posing a serious long-term health risk. The most promising direction of research would be to investigate the expression of the *DPP4* gene.

## Figures and Tables

**Table 1:** Indications for allogeneic HSCT.

Diagnosis	Number (%), *N* = 27
Neoplastic diseases	18 (67)
Acute lymphoblastic leukemia	11 (41)
Acute myeloblastic leukemia	4 (15)
Chronic myelocytic leukemia	1 (4)
Myelodysplastic syndrome	1 (4)
Juvenile myelomonocytic leukemia and acute myeloblastic leukemia	1 (4)
Non-Neoplastic diseases	9 (33)
Severe aplastic anemia	4 (15)
Chronic granulomatous disease	3 (11)
Autoimmune lymphoproliferative syndrome	1 (4)
Hyper IgM syndrome	1 (4)

**Table 2:** Conditioning regimens.

Conditioning Type	Regimen	Number (%), *N* = 27
Non-myeloablative	CyATG	3 (11)
	FluCyATG	1 (4)
Myeloablative	Bu or Bux-based	14 (52)
	Treo-based	2 (7)
	TBI-VP	7 (26)

Abbreviations: ATG—anti-thymocyte globulin, Bu—busulfan, Bux—busilvex, Cy—cyclophosphamide, Flu—fludarabine, TBI-VP—total body irradiation–etoposide, Treo—treosulfan.

**Table 3:** The summary of therapeutic interventions in children referred for allogeneic HSCT.

Treatment		Number of Patients, *N* = 27
Time since diagnosis (years)	Neoplastic diseases	median: 1.0, mean: 2.0, range: 0.1–7.0
	Non-neoplastic diseases	median: 1.5, mean: 3.8, range: 0.1–13.0
Chemotherapy before HSCT (*N*, %)		17 (63)
Local radiotherapy (*N*, %)		5 (19): CNS-4 (15), testes-1 (4)
Total body irradiation-12 Gy/6 fractions (*N*, %)		7 (27)
Conditioning regimen based on busulfan or treosulfan (*N*, %)		16 (59)
GvHD prophylaxis (*N*, %)	CsA	4 (15)
	Mtx + CsA	23 (85)
	ATG	20 (74)
Mucositis (*N*, %)		22 (81)
Grade (*N*)		I-7, II-8, III-6, IV-1
Intravenous alimentation due to mucositis (%)		13 (48)
aGvHD (*N*, %)		11 (41)
Localization (%)		Gut-9, liver-27, skin-91
Grade (*N*)		IA-1, IB-4, IIB-1, IIC-3, IIIC-2
Systemic glucocorticoid treatment	*N*, %	19 (70)
	days	median: 3.5, mean: 3.6, range: 0.1–11.0
Time from HSCT to the second assessment (months)		median: 6.3, range: 5.9–19.1
Time from discontinuation of systemic glucocorticoids to the second assessment (months)		median: 3.6, mean: 4.5, range: 0.5–14.0
Time from discontinuation of immunosuppressive treatment to the second assessment (months)		median: 1.6, range: 0.0–9.0
Hematopoietic stem cells donor (*N*, %)		MUD: 16 (59), MSD: 9 (33), MFD: 2 (7)

Abbreviations: (a)GvHD—(acute) graft-versus-host disease, ATG—anti-thymocyte globulin, CNS—central nervous system, CsA—cyclosporine A, Mtx—methotrexate, MFD—matched family donor, MSD—matched sibling donor, MUD—matched unrelated donor.

**Table 4:** Characteristics of children treated with HSCT.

Characteristic	Pre-HSCT *N* = 27	Post-HSCT *N* = 27
Boys/girls (*N*, %)	20(74)/7(26)	
Age (years)	9.7 ± 5.2	10.4 ± 5.0
Height (cm)	134.7 ± 29.8	137.7 ± 27.2
Body mass (kg)	37.4 ± 18.5	37.2 ± 17.4
Waist circumference (cm)	66.9 ± 12.4	66.1 ± 12.4

**Table 5:** Results of laboratory analysis in children with HSCT procedure.

Parameter	Pre-HSCT *N* = 27	Post-HSCT *N* = 27	*p*/p^BH^-Value Pre-HSCT vs. Post-HSCT
Glc(T_0_) (mmol/L)	4.4 ± 0.6	4.5 ± 0.6	0.7/-
Glc(T_60_) (mmol/L)	6.1 ± 1.0	5.9 ±1.7	0.4/-
Glc(T_120_) (mmol/L)	5.5 ± 1.6	5.4 ± 1.1	0.7/-
AUC glc (mmol/L/h)	11.0 ± 1.4	10.9 ± 1.3	0.6/-
TC (mmol/L)	3.3 ± 1.0	3.9 ± 0.9	0.002/0.04
HDL-C (mmol/L)	1.0 ± 0.4	1.3 ± 0.5	0.02/0.4
LDL-C (mmol/L)	1.4 ± 0.9	1.9 ± 0.8	0.05/-
HDL-C/TC	0.3 ± 0.1	0.4 ± 0.1	0.4/-
TG (mmol/L)	1.8 ± 0.7	1.5 ± 0.6	0.3/-
Insulin(T_0_) (mIU/L)	13.7 ± 13.5	11.3 ± 9.5	0.3/-
Insulin(T_60_) (mIU/L)	56.7 ± 56.2	38.1 ± 46.7	0.05/0.9
Insulin(T_120_) (mIU/L)	43.9 ± 56.2	28.6 ± 32.5	0.3/-
AUC insulin (mIU/L/h)	89.6 ± 98.0	60.5 ± 66.4	0.1/-
Leptin(T_0_) (µg/L)	13.9 ± 20.6	11.6 ± 21.8	0.04/0.7
Leptin(T_60_) (µg/L)	16.2 ± 24.4	8.0 ± 15.7	0.0007/0.02
Leptin(T_120_) (µg/L)	16.8 ± 24.4	8.8 ± 19.0	0.001/0.02
AUC leptin (µg/L/h)	30.1 ± 46.2	18.1 ± 35.5	0.001/0.02
Leptin receptor(T_0_) (µg/L)	27.9 ± 27.4	29.3 ± 24.2	0.4/-
Leptin receptor(T_60_) (µg/L)	28.7 ± 29.5	30.4 ± 21.2	0.1/-
Leptin receptor(T_120_) (µg/L)	28.2 ± 31.5	30.7 ± 22.8	0.3/-
AUC leptin receptor (µg/L/h)	57.4 ± 59.2	61.2 ± 44.4	0.1/-
HOMA-IR	2.8 ± 2.8	2.2 ± 2.0	0.4/-
hsCRP (mg/L)	7.7 ± 10.4	7.0 ± 11.2	0.8/-
Insulin resistance (*N*, %)	9 (41)	6 (26)	0.3/-
Dyslipidemia: Abnormal TC (*N*, %)	1 (4)	2(7)	-
Dyslipidemia: Abnormal TG (*N*, %)	17 (63)	14 (52)	0.6/-
Dyslipidemia: Abnormal HDL-C (*N*, %)	18 (64)	10 (36)	1.0/-
Dyslipidemia: Abnormal LDL-C (*N*, %)	1 (4)	0 (0)	-
Dyslipidemia: Any abnormality (*N*, %)	24 (86)	19 (68)	0.001/0.006

Note: “Dyslipidemia: Any abnormality” means at least one abnormal result for: TC (>5 mmol/L), TG (>1.1 mmol/L (age 0–9) or >1.5 mmol/L (age 10–18)), HDL-C (<1 mmol/L), or LDL-C (>3.2 mmol/L).

**Table 6:** Expression of genes associated with lipid metabolism in children undergoing the HSCT procedure.

Gene Symbol	Locus and Affimetrix Code	Pre-HSCT *N* = 27	Post-HSCT *N* = 27	Pre-HSCT vs. Post-HSCT	
				*FC*	*p*/p^BH^-Value
*DPP4*	*2q24.2* 8056222	481.0 ± 1.5	230.7 ± 1.6	2.07	10^−8^/0.0004
*PLAG1*	*8q12.1* 8150881	68.1 ± 1.4	50.6 ± 1.3	1.34	10^−5^/0.04
*SCD*	*10q24.31* 7929816	125.4 ± 1.4	93.1 ± 1.3	1.35	10^−6^/0.01

**Table 7:** Selected correlations between expressions (or its change Δ_mean_ and relative change Δ_mean(rel)_) of genes associated with lipid metabolism and lipid parameters in children treated with HSCT.

Lipid Metabolism Parameter	Gene	Pre-HSCT Gene Expression and Pre-HSCT Lipid Metabolism Parameters	Post-HSCT Gene Expression and Post-HSCT Lipid Metabolism Parameters	Pre-HSCT Gene Expression and Post-HSCT Lipid Metabolism Parameters	Gene Expression Change (Δ_mean_) and Post-HSCT Lipid Metabolism Parameters	Gene Expression Relative Change (Δ_mean(rel)_) and Post-HSCT Lipid Metabolism Parameters (%)
		Spearman’s Correlation Coefficient r and *p*/p^BH^-Value				
TC	*DPP4*	0.48, 0.02/0.06	−0.42, 0.03/0.1	−0.02, 0.9/-	0.02, 0.9/-	−0.42, 0.03/0.1
	*PLAG1*	0.33, 0.1/0.2	0.30, 0.1/0.2	0.30, 0.1/0.3	0.22, 0.3/0.9	0.23, 0.3/0.6
	*SCD*	−0.13, 0.6/0.6	0.05, 0.8/0.8	0.09, 0.7/-	−0.14, 0.5/-	−0.08, 0.7/0.7
HDL-C	*DPP4*	0.23, 0.3/0.9	0.04, 0.9/-	−0.31, 0.1/0.3	−0.18, 0.4/-	−0.15, 0.5/-
	*PLAG1*	0.01, 1.0/-	0.23, 0.3/0.9	0.01, 1.0/-	0.05, 0.8/-	0.03, 0.9/-
	*SCD*	−0.9, 0.7/-	−0.07, 0.7/-	−0.04, 0.9/-	−0.08, 0.7/-	−0.07, 0.7/-
LDL-C	*DPP4*	0.51, 0.01/0.03	−0.46, 0.02/0.06	0.12, 0.6/-	0.08, 0.7/-	0.11, 0.6/-
	*PLAG1*	0.45, 0.03/0.06	−0.37, 0.06/0.1	0.13, 0.5/-	0.14, 0.5/-	0.15, 0.5/-
	*SCD*	−0.10, 0.7/0.7	0.14, 0.5/0.5	0.18, 0.4/-	−0.20, 0.3/0.9	−0.14, 0.5/-

Note: Significant correlates after Benjamini–Hochberg procedure (p^BH^ < 0.05) are bolded. Only rows with at least one significant *p*-value (non-BH corrected) are shown—the whole correlation matrix is provided in Table S7.

**Table 8:** Expression of genes associated with lipid metabolism in children who presented any feature vs. no feature of dyslipidemia after HSCT.

Gene	Any Feature of Dyslipidemia after the HSCT Procedure, Yes (*N* = 19) vs. No (*N* = 8) (Mean ± SD)		*p*/p^BH^-Value
Gene expression before HSCT			
*DPP4*	509.8 ± 170.2	348.1 ± 115.9	0.02/0.06
*PLAG1*	71.4 ± 22.6	69.8 ± 25.1	0.5/0.5
*SCD*	111.3 ± 44.4	142.8 ± 73.6	0.1/0.2
Gene expression after HSCT			
*DPP4*	220.9 ± 111.6	254.9 ± 85.3	0.3/0.6
*PLAG1*	46.9 ± 6.1	57.3 ± 19.5	0.2/0.6
*SCD*	78.4 ± 23.9	76.1 ± 13.3	0.9/0.9
Difference in gene expression (Δ_mean_) between pre-HSCT and post-HSCT status			
*DPP4*	−328.2 ± 204.0	−46.0 ± 166.6	0.002/0.006
*PLAG1*	−31.2 ± 12.6	−12.6 ± 28.1	0.3/0.3
*SCD*	−32.9 ± 38.7	−66.7 ± 72.7	0.1/0.2
Difference in relative gene expression (Δ_mean(rel)_) between pre-HSCT and post-HSCT status (%)			
*DPP4*	−57.1 ± 36.0	−3.8 ± 51.0	0.002/0.006
*PLAG1*	−35.8 ± 24.6	−11.1 ± 36.7	0.052/0.1
*SCD*	−24.5 ± 19.7	−37.7 ± 22.9	0.1/0.1

Significant differences after Benjamini–Hochberg procedure (p^BH^ < 0.05) are bolded.

**Table 9:** The multiple linear regression model predicting the LDL-C concentration after the HSCT procedure.

Variable	Non-Standardized Regression Coefficient ± SEM	*p*-Value
Sex, boys vs. girls	0.24 ± 0.15	0.1
BMI WHO, per 1 unit	0.05 ± 0.04	0.2
*DPP4* expression after HSCT, per 50 units	−0.12 ± 0.07	0.1

Note: The non-standardized regression coefficient refers to the LDL-C concentration in mmol/L. The R^2^_adj_ was 0.21, with *p* equal to 0.04. The model that included age and an indication for the HSCT (malignant vs. nonmalignant) had a lower ability to predict LDL-C after HSCT (R^2^_adj_ = 0.16, *p* = 0.13).

**Table 10:** The multiple logistic regression models predicting the presence of dyslipidemia after the HSCT procedure.

Variable	Model 1		Model 2		Model 3	
	OR (95% CI)	*p*/p^BH^-Value	OR (95% CI)	*p*/p^BH^-Value	OR (95% CI)	*p*/p^BH^-Value
Sex, boys vs. girls	5.46 (0.36–84.10)	0.2/0.6	6.60 (0.27–159.74)	0.2/0.6	8.09 (0.36–182.76)	0.2/0.6
Age after HSCT, per 1 year	0.92 (0.70–1.21)	0.6/-	0.94 (0.69–1.27)	0.7/-	0.97 (0.73–1.30)	0.9/-
Indication for HSCT, non-malignant vs. malignant	0.29 (0.03–3.28)	0.3/0.9	0.34 (0.03–4.63)	0.4/0.9	0.27 (0.02–3.53)	0.3/0.9
BMI WHO, per 1 unit	0.83 (0.55–1.23)	0.4/0.9	0.80 (0.50–1.29)	0.4/0.9	0.77 (0.48–1.25)	0.3/0.9
*DPP4* expression ^†^	1.51 (1.01–2.25)	0.04/0.04	1.45 (1.08–1.95)	0.01/0.03	1.35 (1.05–1.73)	0.02/0.04

^†^ Model 1: *DPP4* expression before HSCT (per increment of 50 units); model 2: *DPP4* Δ_mean_ of expression between pre- and post-HSCT measurement (per increment of 50 units); model 3: Δ_mean(rel)_ of expression between post- and pre-HSCT measurement (per increment of 10%). In each model, the *p*-value in the Hosmer–Lemeshow goodness-of-fit test was 0.1.

## Data Availability

The data sets generated for this study are available upon request from the corresponding author.

## Supplementary Materials

The following are available online at https://www.mdpi.com/article/10.3390/cancers13143614/s1, Table S1: Difference in the expression of 22 significantly changed genes in children undergoing the HSCT procedure, Table S2: Results of laboratory analysis in children with HSCT procedure (44 subjects), Table S3: Results of laboratory analysis in children with HSCT procedure, with focus on the indication for the transplantations (neoplasm or non-neoplasm), Table S4: Anthropometric characteristics in children with HSCT procedure, Table S5: Anthropometric characteristics in children with HSCT procedure and their counterparts from the control groups (44 HSCT subjects), Table S6: Difference in the expression of genes in children undergoing the HSCT procedure, with focus on the indication for the transplantations (neoplasm or non-neoplasm), Table S7: Correlation between the expression (or its change Δ_mean_ and relative change Δ_mean(rel)_) of genes associated with lipid metabolism and its parameters in children undergoing HSCT procedure.
