# Supplementary material for: The Expression of Genes Related to Lipid Metabolism and Metabolic Disorders in Children before and after Hematopoietic Stem Cell Transplantation—A Prospective Observational Study
Source: Cancers (Basel). 2021 Jul 19;13(14):3614. doi: 10.3390/cancers13143614 (PMC8306759; doi:10.3390/cancers13143614)
Supplement: Supplementary file 1 [file cancers-13-03614-s001.zip › cancers-1271498-supplementary.pdf]

Table S1. Difference in the expression of 22 significantly changed genes in children undergoing the HSCT procedure.

Gene symbol	Locus and Affimetrix code	pre-HSCT *N* = 27	post-HSCT *N* = 27	pre-HSCT vs. post-HSCT	
				FC	**p/p^{BH}-Value**
DPP4	*2q24.2* 8056222	8.91 ± 0.56	7.85 ± 0.68	2.07	10^{-8}/0.0004
PLAG1	*8q12.1* 8150881	6.09 ± 0.47	5.66 ± 0.33	1.34	10^{-5}/0.04
SCD	*10q24.31* 7929816	6.97 ± 0.48	6.54 ± 0.33	1.35	10^{-6}/0.01
BPGM	*7q33* 8136341	9.05 ± 1.18	7.97 ± 0.51	2.11	10^{-6}/0.01
AHSP	*16p11.2* 7995237	8.99 ± 2.21	6.50 ± 1.15	5.62	10^{-6}/0.01
GYPA	*4q31.21* 8102998	7.04 ± 1.85	5.18 ± 0.86	3.62	10^{-6}/0.02
NR3C2	*4q31.23* 8103094	6.55 ± 0.58	5.80 ± 0.46	1.68	10^{-6}/0.02
HMBS	*11.q23.3* 7944401	7.77 ± 0.84	7.23 ± 0.52	1.45	10^{-6}/0.02
C1orf228	*1p34.1* 7900999	7.02 ± 0.22	6.74 ± 0.26	1.21	10^{-6}/0.02
MYB	*6q23.3* 8122202	7.78 ± 0.69	7.41 ± 0.35	1.29	10^{-6}/0.02
CA1	*8q21.2* 8151592	10.08 ± 2.24	7.25 ± 1.68	7.14	10^{-5}/0.02
STRADB	*2q33.1* 8047443	10.19 ± 1.14	9.15 ± 0.59	2.05	10^{-5}/0.02
ST13	*22q13.2* 8076272	9.62 ± 0.28	9.20 ± 0.27	1.34	10^{-5}/0.02
-	*2q33.1* 7901336	10.46 ± 1.18	9.30 ± 0.64	2.22	10^{-5}/0.02
ALAS2	*Xp11.21* 8173135	10.35 ± 2.03	8.12 ± 1.44	4.71	10^{-5}/0.03
IFIT1B	*10q23.31* 7929061	7.76 ± 1.94	5.93 ± 1.03	3.55	10^{-5}/0.03
-	*8p* 8146157	4.02 ± 0.57	3.55 ± 0.27	1.39	10^{-5}/0.04
SPIN3	*Xp11.21* 8173181	6.67 ± 0.23	6.39 ± 0.26	1.21	10^{-5}/0.04
TCEA3	*1p36.12* 7913593	7.39 ± 0.49	6.95 ± 0.30	1.35	10^{-5}/0.04
ST13P5	*11p15.1* 7938756	8.40 ± 0.32	8.10 ± 0.27	1.23	10^{-5}/0.04
RNF157	*17q25.1* 8018652	8.52 ± 0.60	7.93 ± 0.47	1.51	10^{-5}/0.04
KIAA0226L	*13q14.1* 7971486	7.35 ± 0.80	8.12 ± 0.61	-1.71	10^{-5}/0.04

The expression of genes is given here as log_2 of the raw expression data.

Table S2. Results of laboratory analysis in children with HSCT procedure (44 subjects).

Parameter	pre-HSCT $N = 44$	post-HSCT $N = 27$	p-Value
Glc[T_0] (mmol/L)	4.48±0.59	4.45±0.59	0.69
Glc[T_{60}] (mmol/L)	6.38±1.06	5.91±0.91	0.37
Glc[T_{120}] (mmol/L)	5.59±1.49	5.4±1.05	0.69
AUC glc (mmol/L/h)	11.4±1.51	10.85±1.32	0.57
TC (mmol/L)	3.45±1.01	3.92±0.89	**0.002**
HDL-C (mmol/L)	1.07±0.36	1.36±0.51	**0.016**
LDL-C (mmol/L)	1.62±0.92	1.86±0.8	**0.045**
HDL-C/TC	0.32±0.11	0.35±0.12	0.37
TG (mmol/L)	1.68±0.68	1.54±0.61	0.25
hsCRP (mg/L)	9.8±13.35	6.99±11.22	0.75
Insulin[T_0] (mIU/L)	14.4±13.3	11.28±9.47	0.31
Insulin[T_{60}] (mIU/L)	57.88±50.59	38.09±46.67	**0.045**
Insulin[T_{120}] (mIU/L)	42.3±48.93	28.56±32.54	0.30
AUC insulin (mIU/L/h)	91.41±85.2	60.48±66.38	0.12
Leptin[T_0] (µg/L)	17.56±27.25	11.3±21.38	**0.037**
Leptin[T_{60}] (µg/L)	17.82±26.41	10.31±19.17	**<0.001**
Leptin[T_{120}] (µg/L)	17.66±25.63	11.36±22.8	**0.001**
AUC leptin (µg/L/h)	36.12±53.75	21.88±17.43	**0.001**
Leptin receptor[T_0] (µg/L)	24.38±22.99	28.89±23.77	0.43
Leptin receptor[T_{60}] (µg/L)	25.16±24.3	28.86±21.04	0.11
Leptin receptor[T_{120}] (µg/L)	24.86±25.91	29.13±22.51	0.26
AUC leptin receptor (µg/L/h)	50.25±48.72	58.13±43.94	0.14
HOMA-IR	2.98±2.8	1.82±1.97	0.40
Insulin resistance (*N*, %)	14(31.82%)	5(27.78%)	0.27

Table S3. Results of laboratory analysis in children with HSCT procedure with focus on the indication for the transplantations (neoplasm or non-neoplasm).

Parameter	pre-HSCT			post-HSCT		
	neoplasm $N = 18$	non-neoplasm $N = 9$	*p*-Value	neoplasm $N = 18$	non-neoplasm $N = 9$	*p*-Value
Glc[T_0] (mmol/L)	4.4±0.6	4.5±0.5	0.5	4.4±0.4	4.6±0.9	0.4
Glc[T_{60}] (mmol/L)	6.2±1.1	6.3±0.8	0.9	5.8±0.7	6.0±1.2	0.6
Glc[T_{120}] (mmol/L)	5.5±1.5	5.5±1.9	0.9	5.7±0.7	5.0±1.4	0.2
AUC glc (mmol/L/h)	11.0±1.4	11.3±1.5	0.7	10.9±0.9	10.8±1.8	1.0
TC (mmol/L)	3.2±1.0	3.5±1.2	0.5	3.9±1.0	3.9±0.6	1.0
HDL-C (mmol/L)	1.1±0.4	0.9±0.3	0.3	1.4±0.5	1.4±0.5	0.9
LDL-C (mmol/L)	1.2±0.9	1.9±1.0	0.1	1.9±0.9	1.8±0.6	0.7
HDL-C/TC	0.4±0.1	0.3±0.1	0.1	0.4±0.1	0.3±0.1	0.8
TG (mmol/L)	1.9±0.8	1.5±0.6	0.2	1.4±0.5	1.8±0.7	0.2
Insulin[T_0] (mIU/L)	12.6±9.8	15.5±19.1	0.6	8.1±4.7	17.2±13.3	0.1
Insulin[T_{60}] (mIU/L)	44.3±25.4	78.4±87.8	0.3	31.1±28.6	31.1±68.8	0.3
Insulin[T_{120}] (mIU/L)	28.6±22.5	75.5±89.1	0.2	21.0±13.9	42.1±49.9	0.2
AUC insulin (mIU/L/h)	64.1±33.6	149.2±165.8	0.3	46.3±35.4	85.3±98.9	0.2
Leptin[T_0] (µg/L)	12.3±20.5	15.5±21.0	0.7	10.3±19.5	13.2±25.6	0.8
Leptin[T_{60}] (µg/L)	15.1±25.6	16.9±21.4	0.9	8.1±17.7	15.4±22.8	0.4
Leptin[T_{120}] (µg/L)	13.9±23.9	22.3±25.4	0.4	8.9±21.5	17.0±26.5	0.4
AUC leptin (µg/L/h)	23.7±44.5	45.5±49.0	0.4	17.3±38.3	32.4±50.2	0.4
Leptin receptor[T_0] (µg/L)	19.8±9.9	43.7±41.7	0.1	28.1±22.6	30.4±27.2	0.8
Leptin receptor[T_{60}] (µg/L)	20.1±10.3	52.8±46.5	0.1	27.8±19.2	30.8±25.1	0.7
Leptin receptor[T_{120}] (µg/L)	19.6±12.9	49.2±48.4	0.1	27.8±19.5	31.4±28.2	0.7
AUC leptin receptor (µg/L/h)	39.8±20.6	106.4±92.8	0.1	56.1±40.2	61.7±52.4	0.8
HOMA-IR	2.0±2.2	2.5±3.6	0.6	1.3±1.0	2.7±2.9	0.2
hsCRP (mg/L)	4.8±4.3	12.5±15.4	0.2	7.3±12.2	6.4±9.9	0.8
Insulin resistance (*N*, %)	5 (28)	4 (44)	0.7	2 (11)	4 (44)	0.1

Dyslipidemia: abnormal TC (*N*, %)	0 (0)	1 (11)	0.7	2 (11)	0 (0)	0.5
Dyslipidemia: abnormal TG (*N*, %)	12 (67)	5 (56)	0.3	7 (39)	7 (78)	0.1
Dyslipidemia: abnormal HDL-C (*N*, %)	11 (61)	7 (78)	0.7	7 (39)	3 (33)	0.7
Dyslipidemia: abnormal LDL-C (*N*, %)	0 (0)	1 (11)	0.4	0 (0)	0 (0)	1.0
Dyslipidemia: any abnormality (*N*, %)	15 (83)	9 (100)	1.0	11 (61)	8 (89)	0.4

"Dyslipidemia: any abnormality" means at least one abnormal of: TC (>5 mmol/L), TG (>1.1 mmol/L (age 0-9) or >1.5 mmol/L (age 10-18)), HDL-C (<1 mmol/L) and LDL-C (>3.2 mmol/L).

Table S4. Anthropometric characteristics in children with HSCT procedure**.**

	**pre-HSCT** ***N*** **= 27**	**post-HSCT** ***N*** **= 27**	**p/p^{BH}-Value pre-HSCT vs. post-HSCT**
BMI WHO (kg/m^2)	19.0 ± 4.2	18.4 ± 3.5	0.2/-
BMI WHO percentile	61.3 ± 31.1	53.0 ± 35.4	0.2/-
TBW (L)	27.7 ± 19.8	22.4 ± 9.1	0.6/-
ECW (L)	13.7 ± 14.8	10.1 ± 4.0	0.2/-
BF[kg]	11.8 ± 17.9	7.5 ± 4.9	0.6/-
BF[%]	18.7 ± 14.8	17.7 ± 6.9	0.7/-
LBM[kg]	34.0 ± 16.1	30.7 ± 12.6	0.8/-

Table S5. Anthropometric characteristics in children with HSCT procedure and their counterparts from the control groups (44 HSCT subjects).

**Parameters**	**pre-HSCT** ***N*** **= 44**	**post-HSCT** ***N*** **= 27**	**p-Value pre-HSCT vs. post-HSCT**
BMI WHO (kg/m^2)	19.02±4.21	18.35±3.47	0.16
BMI WHO percentile	61.26±31.12	53±35.41	0.17
TBW (L)	27.65±19.83	22.39±9.07	0.64
BF[kg]	11.77±17.94	7.48±4.87	0.61
BF[%]	18.65±14.84	17.68±6.88	0.73
LBM[kg]	33.95±16.18	30.65±12.57	0.82
ECW (L)	13.71±14.84	10.05±3.96	0.16

Table S6. Difference in the expression of genes in children undergoing the HSCT procedure with focus on the indication for the transplantations (neoplasm or non-neoplasm).

**Gene symbol**	**Locus and Affimetrix code**	**pre-HSCT**				**post-HSCT**			
		**neoplasm** ***N*** **= 18**	**non-neoplasm** ***N*** **= 9**	***FC***	**p/p^{BH}-Value**	**neoplasm** ***N*** **= 18**	**non-neoplasm** ***N*** **= 9**	***FC***	**p/p^{BH}-Value**
DPP4	*2q24.2* 8056222	8.69±0.78	8.63±0.73	1.04	1.0/1.0	7.95±0.78	8.24±0.50	-1.22	0.3/1.0

Table S7. Correlation between the expression (or its change Δ_{mean} and relative change $\Delta_{mean(rel)}$) of genes associated with lipid metabolism and its parameters in children undergoing HSCT procedure.

Lipid metabolism parameter	Gene	Pre-HSCT gene expression and pre-HSCT lipid metabolism parameters	Post-HSCT gene expression and post-HSCT lipid metabolism parameters	Pre-HSCT gene expression and post-HSCT lipid metabolism parameters	Gene expression change (Δ_{mean}) and post-HSCT lipid metabolism parameters	Gene expression relative change ($\Delta_{mean(rel)}$) and post-HSCT lipid metabolism parameters (%)
		Spearmann's correlation coefficient r and p/p^{BH}-Value				
TC	DPP4	0.48, 0.02/0.06	-0.42, 0.03/0.1	-0.02, 0.9/-	0.02, 0.9/-	-0.42, 0.03/0.1
	PLAG1	0.33, 0.1/0.2	0.30, 0.1/0.2	0.30, 0.1/0.3	0.22, 0.3/0.9	0.23, 0.3/0.6
	SCD	-0.13, 0.6/0.6	0.05, 0.8/0.8	0.09, 0.7/-	-0.14, 0.5/-	-0.08, 0.7/0.7
HDL-C	DPP4	0.23, 0.3/0.9	0.04, 0.9/-	-0.31, 0.1/0.3	-0.18, 0.4/-	-0.15, 0.5/-
	PLAG1	0.01, 1.0/-	0.23, 0.3/0.9	0.01, 1.0/-	0.05, 0.8/-	0.03, 0.9/-
	SCD	-0.9, 0.7/-	-0.07, 0.7/-	-0.04, 0.9/-	-0.08, 0.7/-	-0.07, 0.7/-
LDL-C	DPP4	**0.51, 0.01/0.03**	-0.46, 0.02/0.06	0.12, 0.6/-	0.08, 0.7/-	0.11, 0.6/-
	PLAG1	0.45, 0.03/0.06	-0.37, 0.06/0.1	0.13, 0.5/-	0.14, 0.5/-	0.15, 0.5/-
	SCD	-0.10, 0.7/0.7	0.14, 0.5/0.5	0.18, 0.4/-	-0.20, 0.3/0.9	-0.14, 0.5/-
TG	DPP4	-0.21, 0.3/0.9	0.03, 0.9/-	-0.02, 0.9/-	0.13, 0.5/-	0.18, 0.4/-
	PLAG1	-0.14, 0.5/-	-0.17, 0.4/-	0.07, 0.7/-	0.05, 0.8/-	0.04, 0.8/-
	SCD	-0.07, 0.7/-	-0.09, 0.7/-	-0.13, 0.5/-	0.06, 0.8/-	0.06, 0.8/-
HDL-C/TC	DPP4	-0.13, 0.6/-	0.32, 0.1/0.2	-0.31, 0.1/0.3	-0.21, 0.3/0.9	-0.18, 0.4/-
	PLAG1	-0.30, 0.2/0.6	0.42, 0.03/0.1	-0.01, 1.0/-	0.03, 0.9/-	0.01, 1.0/-
	SCD	-0.08, 0.7/-	-0.11, 0.6/0.6	0.01, 0.9/-	-0.08, 0.7/-	-0.09, 0.7/-
AUC Glc	DPP4	0.11, 0.6/-	-0.11, 0.6/-	0.34, 0.1/0.3	0.34, 0.1/0.3	0.21, 0.3/0.9
	PLAG1	0.35, 0.1/0.3	0.17, 0.4/-	0.11, 0.6/0.6	0.05, 0.8/-	0.05, 0.8/-
	SCD	-0.01, 1.0/-	-0.04, 0.8/-	-0.26, 0.2/0.4	0.16, 0.5/-	0.15, 0.5/-
AUC insulin	DPP4	0.43, 0.06/0.2	-0.25, 0.3/0.6	0.42, 0.06/0.2	0.25, 0.3/0.6	0.21, 0.4/0.8
	PLAG1	0.41, 0.07/0.2	-0.28, 0.2/0.6	0.10, 0.7/-	0.13, 0.6/0.6	0.16, 0.5/0.8
	SCD	0.07, 0.8/0.8	0.19, 0.4/0.6	-0.13, 0.6/-	0.31, 0.2/0.6	0.33, 0.1/0.3
AUC leptin	DPP4	0.42, 0.07/0.2	-0.16, 0.5/-	0.32, 0.2/0.6	0.18, 0.4/-	0.02, 0.9/-
	PLAG1	0.26, 0.3/0.3	-0.25, 0.3/0.9	0.13, 0.6/0.8	0.16, 0.5/-	0.17, 0.5/-
	SCD	-0.36, 0.1/0.2	-0.13, 0.6/-	-0.18, 0.4/0.8	0.20, 0.4/-	0.17, 0.4/-
AUC leptin receptor	DPP4	-0.14, 0.5/0.6	0.13, 0.5/-	-0.29, 0.2/0.6	-0.15, 0.5/-	-0.01, 1.0/-
	PLAG1	0.24, 0.3/0.6	0.08, 0.7/-	-0.06, 0.8/-	0.05, 0.8/-	0.08, 0.7/-
	SCD	0.28, 0.2/ 0.6	0.11, 0.6/-	0.09, 0.7/-	-0.03, 0.9/-	-0.01, 1.0/-

Significant correlates after Benjamini-Hochberg procedure (p^{BH} <0.05) are bolded.

PLAG1	*8q12.1* 8150881	6.06±0.41	6.38±0.40	-1.25	0.01/0.3	5.80±0.32	5.65±0.43	1.11	0.9/1.0
SCD	*10q24.31* 7929816	6.82±0.63	6.83±0.54	-1.00	0.4/0.8	6.46±0.63	6.55±0.46	-1.06	0.7/1.0

The expression of genes is given here as log_2 of the raw expression data.
